# Motor and Cognitive Modulation of a Single Session of Transcutaneous Auricular Vagus Nerve Stimulation in Post Stroke Patients: A Pilot Study

**DOI:** 10.1109/OJEMB.2023.3268011

**Published:** 2023-04-21

**Authors:** M. Colombo, S. Aggujaro, N. Lombardi, A. Pedrocchi, F. Molteni, Eleonora Guanziroli

**Affiliations:** Villa Beretta Rehabilitation Center Costa Masnaga – Lecco 220196 23845 Como Italy; Nearlab, Department of Electronics, Informatics and BioengineeringPolitecnico di Milano 18981 20133 Milan Italy

**Keywords:** Cognitive functions, ipsilesional upper limb motor functions, modulation, stroke rehabilitation, transcutaneous vagus nerve stimulation

## Abstract

*Objective*: The aim of the present study is to explore whether a single session of transcutaneous Vagus Nerve Stimulation (tVNS) can enhance the ipsilesional, and contralesional upper limb motor functions as well as cognitive functions in stroke patients. The effects of the stimulation were evaluated through two different tasks: the box and blocks test (BB), indexing manual dexterity, and the Go/No-go task, a visuomotor paradigm used to assess both motor readiness and response inhibition. Tests were administered without tVNS, during tVNS and during sham tVNS. *Results*: The BB showed a statistical difference for both contralesional side (p = 0.05) between Basal-Real condition (p = 0.042) and ipsilesional side (p = 0.001) between Basal-Real (p = 0.008) and for Real-Sham (p = 0.005). Any statistical difference was found for the mean latencies in the three conditions of the Go/No-go test. *Conclusion*: A single session of tVNS seems to improve upper limb motor functions but not cognitive functions in post-stroke patients, despite a positive trend was detected.

## I. Introduction

The prevalence of upper limb impairment following stroke is approximately 50–80% in the acute phase [1] and 40–50% in the chronic phase [2]. Since deficits may involve both the contralesional and ipsilesional upper limb, patients may experience reduced ability to perform everyday activities.

Clinical intervention in the acute phase after stroke is primarily focused on sensorimotor recovery of the contralesional arm that is influenced by the quantity and quality of movements performed during training [1].

Despite the main functional limitations in post-stroke patients are related to motor deficits of the contralateral side of the body [3], stroke limits also the motor functions of the ipsilesional side, which is generally considered the non-affected side [4]. Sensory motor functions of the ipsilesional upper limb is usually regained one month after stroke, but with worse capacity/performance than healthy subjects and impaired reaction time persisting one year after stroke [5].

Not only motor, but also cognitive impairments are frequently observed after stroke. In particular, global cognition, executive dysfunction, and impaired memory are associated with motor impairments of lower and upper limbs [6]. The prevalence of post-stroke neurocognitive disorders ranges from 7 to 67.3%, depending on stroke type (ischemic or hemorrhagic), the presence of pre-stroke dementia, and temporal distance from the acute event [7].

Recognizing the impact of stroke on motor and cognitive functions is an important step for implementing rehabilitation programs that encompass both motor and cognitive aspects.

Novel technologies, based on robotics [8], neuromuscular electrical stimulation [9], non-invasive brain stimulation [10] or virtual reality [11] have been proposed as adjuvant tools for motor and cognitive rehabilitation.

The diffusion of non-invasive brain stimulation techniques offers the possibility to directly interact with brain activity and related functions. These techniques are a promising tool to shed light on brain dynamics underlying human behavior in neurological patients and may be applied in the rehabilitation field to restore maladaptive plasticity characterizing several clinical conditions.

Transcutaneous auricular vagus nerve stimulation (tVNS) is a novel neuromodulation technique that has recently emerged as an alternative method to invasive vagus nerve stimulation (iVNS) [12]. Despite being a minimally invasive procedure, the surgery of iVNS is highly risky due to the location of the implantation, with electrode placement requiring dissection of the vagus nerve from the carotid artery [13], [14]. Therefore, such potential adverse events limit the applicability of this intervention.

Instead, the non-invasive adaptation of iVNS technique (tVNS) is attracting growing interest as a possible intervention for a wide range of neurological disorders [15], as it may contribute to the enhancement of neural plasticity and thus support functional recovery in pathological conditions [16]. Moreover, tVNS has minimal side effects and is inexpensive and portable, thus being potentially applicable as an adjuvant treatment also in combination with other rehabilitation interventions.

tVNS was originally used as an alternative treatment option for epilepsy and depression. Recent studies [17] showed that vagus nerve stimulation, paired with rehabilitation, is a novel treatment option also for people with long-term moderate-to-severe arm impairment after ischemic stroke. Additionally, it may be used to modulate cognitive functions, in particular for emotion recognition, fear extinction, cognitive control, and attention, even if no univocal effects have been found across cognitive domains [18], [19], [20].

Several studies focusing on tVNS effects on memory in both healthy subjects and patients with Alzheimer's disease showed modulation effects across different types of memory in non-demented patients after both short-term and chronic tVNS application [21].

Finally, a recent meta-analysis [22] investigating the effects of tVNS on cognition in healthy individuals, including executive functions, concluded that there is some evidence supporting the theory that tVNS could play a role in improving response inhibition in higher cognitive demanding tasks.

Starting from these literature evidences, tVNS should improve upper limb and cognitive function. In particular, the aim of the present study was to explore whether a single session of tVNS can enhance the upper limb motor functions and cognitive functions in a sample of ten stroke patients. The effects of the stimulation were evaluated using two different tasks: the box and blocks test (BB), indexing manual dexterity, and the Go/No-go task, a visuomotor paradigm used to assess both motor readiness and response inhibition. BB and Go/NoGo tests were chosen because these tests are easy, quick to administer, without learning effects with the possibility to administer these tests more time in short period.

## II. Results

Ten post-stroke patients were evaluated. Six males and four females with mean age of 61.9 ± 11.22 years. All patients were right-handed before the event. Population characteristics are presented in Table 1. The study was approved by Comitato Etico della Sezione “IRCCS Fondazione Don Carlo Gnocchi” (n 25/2022, September 25^th^, 2022).

**TABLE 1:** Population Characteristics

ID	Age	Sex	Education years	Ischemic Hemorrhagic	Acute Chronic	Lesion site
1	48	M	18	I	A	right F-P
2	55	M	8	H	C	right P-O
3	61	M	8	I	A	left pontine
4	45	F	18	I	C	right T-P, right P-O
5	60	M	13	H	A	left F + deep lesion (IC)
6	57	F	8	H	C	left deep (CL)
7	72	F	8	I	A	left F-P
8	68	F	5	H	A	right deep (CP)
9	79	M	18	I	A	medulla
10	74	M	13	H	A	left F

Table I: demographic and clinical variables for patients enrolled. (P, parietal lobe; O, occipital lobe; F, frontal lobe; IC, internal capsule; CL, capsule-lenticular; CP, capsule-thalamus)

Global Motor Evaluation in terms of body functions (Table 2) and body activities (Table 3) following ICF classification was performed on subjects enrolled.

**TABLE 2:** Motor Evaluation (Body Function)

ID	Pain			Modified Ashworth Scale										
	Shoulder	Elbow	Wrist	Shoulder				Elbow		Wrist		Finger	
				Add	Abd	F	E	F	E	F	E	F	E
1	8	1	1	3	1	2	2	3	3	0	3	0	3
2	0	0	0	2	2	2	0	2	2	0	0	0	0
3	0	0	0	0	0	0	0	0	0	0	0	0	0
4	5	2	2	1	0	0	0	0	1	0	1	0	1
5	0	0	0	1	0	0	0	0	1	0	1	0	0
6	0	0	0	0	0	0	0	0	1	0	1	0	1
7	0	0	0	0	0	0	0	0	0	0	0	0	0
8	0	0	0	0	0	0	0	0	0	0	0	0	0
9	0	0	0	0	0	0	0	0	0	0	0	0	0
10	1	0	0	1	0	0	0	0	1	0	1	0	1
**Median**	0	0	0	1	0	0	0	0	1	0	1	0	0
**IQR**	0.75	0	0	1	0	0	0	0	1	0	1	0	1

Table II: Clinical scales of body function ICF domain (Adductor (Add), Abductor (Abd), F (Flexors), E (Extensor))

**TABLE 3:** Motor Evaluation (Body Activities)

ID	Motricity Index		Fugl-Meyer				TCT	ARAT		9HPT	
	Contra	Ipsi	Tot UL	Sen	pRom	Pain		Contra	Ipsi	Contra	Ipsi
1	1	100	4	1	12	7	48	0	57	ne	26
2	85	100	56	10	22	24	100	56	57	45	29
3	56	100	40	9	20	18	87	29	57	ne	38
4	35	100	22	5	12	12	74	3	57	ne	26
5	15	100	4	12	24	24	61	3	57	ne	34
6	65	100	31	8	20	23	48	13	57	ne	25
7	92	100	62	12	22	24	100	57	57	36	29
8	71	100	57	7	19	21	74	47	57	73	27
9	92	100	56	12	24	24	61	55	57	35	112
10	48	100	14	12	21	20	61	3	57	ne	32
**Median**	61	100	36	10	21	22	68	21	57	0	29
**IQR**	43	0	40	5	3	6	23	50	0	56	7

Table III: Clinical scales of body activities ICF domain (Motricity Index (MI) for Contra and Ipsilesional upper limb, Fugl-Meyer, Trunk Control Test (TCT), Action Reaction Arm Test (ARAT); 9 Hole Peg Test (9HPT) for the contra and ipsilesional upper limb. For 9NPT ne = not executable.

Patients were characterized by absence of pain at all districts considered and limited spasticity was present only at shoulder adductor, wrist, and elbow flexors.

The ipsilesional side of patients enrolled into the study was characterized by:

• no deficits in upper extremity functional mobility (Motricity Index (MI) = 100/100);
• no deficits in coordination and dexterity (Action Research Arm Test (ARAT) = 57/57);
• fingers dexterity measured with 9Hole Peg Test (9HPT) was 29s (normative value = 20.87s).

The contralesional side was characterized by:

• functional mobility evaluated with MI equal to 61/100;
• Fugl-Meyer (FM) motor upper limb section was 36/66 and sensibility section was 10/24;
• no limitation in passive ROM (FM = 21/24) and no pain (FM = 22/24);
• the coordination and dexterity measured with ARAT was 21/57;
• fingers dexterity (9HPT) was 41/20.87 s;
• a deficit in trunk control was measured (Trunk Control Test (TCT) = 68/100).
• All patients were right-handed.

The neuropsychological screening results (Table 4) is presented as Raw Score (RS), and corrected (CS) scores in order to overcome the influence of one or more of the following variables: age, gender, years of education. The CS obtained were then converted into an Equivalent Score (ES), with a score of 0 meaning that the performance was less than 5% of the population (deficit) and a score of 4 indicating a score equal to or above the population mean. 1, 2 and 3 were intermediate scores with a quasi-interval distribution. In particular 1 represented a borderline performance, 2 and 3 a performance within the normal range.

**TABLE 4:** Cognitive Evaluation

ID	MMSE		FAB			DSF			DSB		
	RS	CS	RS	CS	ES	RS	CS	ES	RS	CS	ES
1	29	26.2	17	16.7	3	8	7.60	4	5	4.5	4
2	30	28	18	17.9	4	5	5.04	2	4	4.1	3
3	29	27	14	13.6	1	7	6.77	4	5	4.66	4
4	29	26.2	18	16.7	4	9	8.53	4	6	5.43	4
5	28	25.2	14	13.5	1	7	6.92	4	7	6.87	4
6	28	26	15	14.3	1	4	3.83	0	4	3.79	3
7	26	23.7	11	10.7	0	6	5.96	4	3	2.87	1
8	24	21.9	9	10.3	0	3	3.51	0	2	2.64	0
9	28	26.3	15	15	2	5	5.30	3	4	4.16	3
10	26	23.7	11	10.7	0	6	5.99	4	3	2.87	1
**Median**	28.00	26.10	14.50	13.95	1.00	6.00	5.98	4.00	4.00	4.13	3.00
**IQR**	2.50	2.20	4.75	4.88	2.50	2.00	1.78	1.75	1.75	1.52	2.5

Table IV: Neuropsychological scales Mini Mental State Examination (MMSE), Frontal Assessment Battery (FAB), Digit Span Forward (DSF), Digit Span Backward (DSB), Raw Score (RS), Correct Score (CS), Equivalent Score (ES).

Concerning the global cognitive functioning index (MMSE), all patients were within the normal range; regarding the executive domain (FAB), six patients out of ten obtained scores below the normal range. Verbal working memory and attention (DSF) was preserved in eight out of ten patients, while cognitive control (DSB) was preserved in seven out of ten patients.

*Stimulation Effect: Box and Blocks*

**Figure 1.**
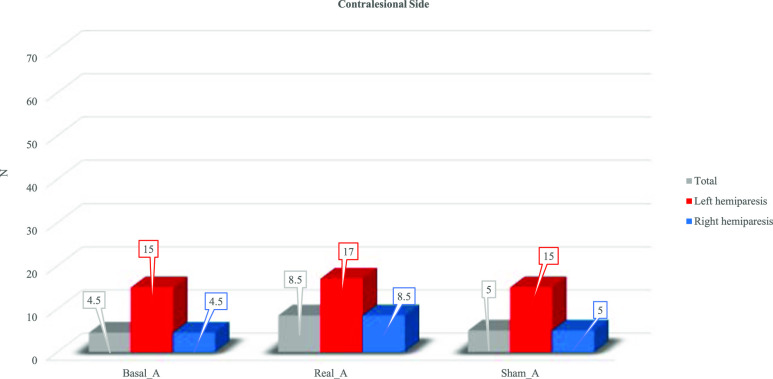
Median values of Box and Blocks test for the contralesional side for the whole population (grey) and for left hemiparetic patients (red) and right hemiparetic patients (blue).

**Figure 2.**
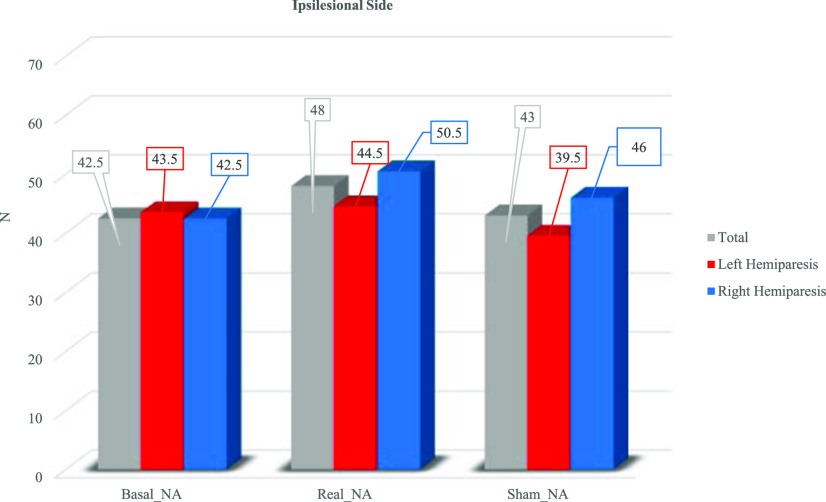
Median values of Box and Blocks test for the ipsilesional side for the whole population (grey) and for left hemiparetic patients (red) and right hemiparetic patients (blue).

Figs. 1 and 2 present the median values obtained at the BB without stimulation (Basal), with stimulation (Real) and sham stimulation (Sham) for the contralesional side (Fig. 1) and for the ipsilesional one (Fig. 2).

For the whole population, the median value of BB performed with the contralesional side was 4.50 without stimulation, 8.50 with tVNS and 5 in sham condition. For patients with left hemiparesis the median value was 15 without stimulation, 17 with tVNS and 15 in sham condition. For patients with right hemiparesis the median value was 4.5 without stimulation, 8.5 with tVNS and 5 in sham condition.

Data for the ipsilesional side for the whole population of BB was 42.50 without stimulation, 48 with tVNS and 43 in sham condition. For patients with left hemiparesis the median value was 43.5 without stimulation, 44.5 with tVNS and 39.5 in sham condition. For patients with right hemiparesis the median value was 42.5 without stimulation, 50.5 with tVNS and 46 in sham condition.

For the whole population, Friedman analysis showed a statistical difference for both contralesional (χ^2^ = 6.0, 2 DOF, p = 0.05) and ipsilesional side (χ^2^ = 14.6, 2 DOF, p = 0.001). Post hoc analysis using Wilcoxon signed-rank test showed a statistical difference for the contralesional side comparing Basal-Real condition (Z = −2.03, p = 0.042) and no differences were found for Basal-Sham (Z = 0.71, p = 0.713) and Real-Sham (Z = −1.76, p = 0.078). For the ipsilesional side a statistical difference between Basal-Real (Z = −2.67, p = 0.008) and for Real-Sham (Z = −2.81, p = 0.005) was found. No differences between Basal-Sham (Z = −1.08, p = 0.282) were found.

Friedman analysis for patients with left hemiparesis showed no statistical differences for both contralesional (χ^2^ = 3.0, 2 DOF, p = 0.223) and ipsilesional side (χ^2^ = 4.93, 2 DOF, p = 0.085).

Friedman analysis for patients with right hemiparesis showed no statistical differences for the contralesional side (χ^2^ = 3.82, 2 DOF, p = 0.148) while a statistical difference was found for the ipsilesional side (χ^2^ = 10.17, 2 DOF, p = 0.006). Post hoc analysis using Wilcoxon signed-rank test conducted on the ipsilesional side showed a statistical difference comparing Basal-Real condition (Z = −2.20, p = 0.028), while no differences were found for Basal-Sham (Z = −1.63, p = 0.102) and Real-Sham (Z = −1.83, p = 0.068).

*Stimulation Effect: Go/No-go*

Table 5 presents the results of Go/No-go test in the three conditions: without stimulation (Basal), with stimulation (Real) and sham stimulation (Sham).

**TABLE 5:** Go/No-Go Test

	Basal				Real				Sham			
ID	mn-RT	md-RT	Err	Omi	mn-RT	md-RT	Err	Omi	mn-RT	md-RT	Err	Omi
1	456	440	1	0	531	519	1	0	432	396	1	0
2	530	518	0	0	444	459	1	0	510	501	1	0
3	453	444	5	0	444	440	3	0	510	516	3	0
4	612	587	0	0	519	455	0	1	600	619	0	1
5	557	576	0	0	551	547	1	0	576	564	1	0
6	854	837	0	0	830	833	0	0	779	806	0	0
7	687	698	1	1	490	466	5	0	508	488	1	2
8	885	885	0	1	477	464	2	1	477	444	3	0
9	547	539	0	0	569	566	2	0	628	651	1	0
10	687	698	1	1	745	762	1	0	730	749	2	0

Table V: Go/No-go test without stimulation (Basal), with stimulation (Real) and sham stimulation (Sham). mn-RT, mean reaction time; md-RT, median reaction time; Err, errors; Omi, omission

**Figure 3.**
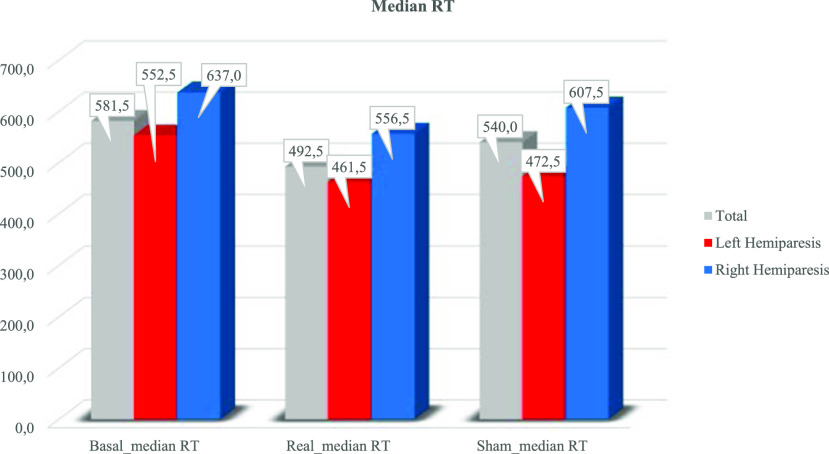
Median values of Go/No-go test (latencies) across the three conditions (T0 versus Real versus Sham) for the entire population (grey), left (red) and right (blue) hemiparetic patients.

Fig. 3presents the median values of Go/No-go test (latencies) across the three conditions (Basal, Real, Sham) for the entire population and for left and right hemiparetic patients.

**Figure 4.**
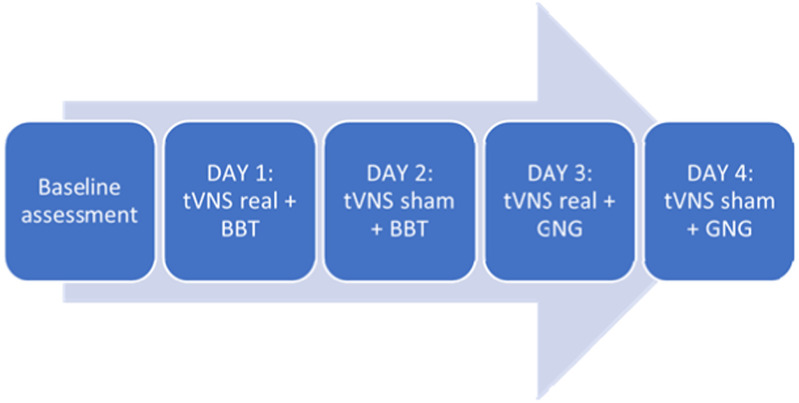
Schematic representation of the experimental design.

For the whole population, any statistical difference was found when comparing the mean latencies (Friedman test: χ^2^ = 2.21, 2 DOF, p = 0.332). A general trend in reduced reaction times was observed comparing Basal-Real condition, although no statistical difference was reached. Seven out of ten patients registered faster response times under real stimulation. Similar findings emerged when comparing Real-Sham stimulation: mean reaction times in six patients were faster than under sham conditions. Any statistical difference was found when comparing the number of errors (Friedman test: χ^2^ = 4.14, 2 DOF, p = 0.124) and the number of omissions (Friedman test: χ^2^ = 0.15, 2 DOF, p = 0.93) under the three conditions.

Friedman analysis showed no statistical differences in the mean latencies for patients with left hemiparesis (χ^2^ = 3.6, 2 DOF, p = 0.165) and with right hemiparesis (χ^2^ = 1.3, 2 DOF, p = 0.513).

## III. Discussion

tVNS is a neuromodulation therapy based on the stimulation of the vagus nerve through the most external part of the ear. The impulses sent reach the neural centers and, in turn, generate corresponding nervous activity [23]. Anatomical studies of the ear suggest that the tragus, concha, and cymba concha are the places on the human body where there are cutaneous afferent vagus nerve distributions [24], therefore stimulation of these afferent fibers should produce therapeutic effects that are similar to those of iVNS [25].

tVNS has been widely used for the clinical treatment of many diseases including epilepsy, drug-refractory depression, pain, and chronic tinnitus [26], [27], and despite the underlying mechanisms are still not fully understood, positive effects have been recently showed also for the treatment of motor impairments after stroke [28]. The effects of tVNS on the activity of the central nervous system are widespread and seem to be mediated by different neurotransmitter systems, including norepinephrine, gamma-aminobutyric acid, dopamine, and serotonin, previously shown to be susceptible to this stimulation [29], [30] and are thought to have a pivotal role in the pathophysiology of several neurological and psychiatric conditions, such as epilepsy [31] and affective disorders [32]. In particular, tVNS may activate the neurons in the nucleus basalis neuron and locus coeruleus, which in turn leads to the release of acetylcholine and norepinephrine in the cerebral cortex. The release of neurotransmitters enhances the synaptic plasticity and the reorganization of cortical networks which ultimately improves motor function [33].

Considering the sensorimotor system, there is a growing number of studies reporting behavioral and electrophysiological effects of tVNS on motor functions [34], [35]. Changes in corticospinal excitability using a paired-pulse TMS protocol were found with a significant increase of short-interval intracortical inhibition in the right motor cortex following 1-hour of tVNS application [36]. Additionally, behavioral and electrophysiological modulation of the readiness potential were found in the right hemisphere, suggesting that tVNS is able to modulate the activity of the motor system contralateral to the stimulated ear [37]. Given the potential application of the technique as an adjuvant treatment of motor disorders following acquired brain injury, the effects of tVNS on motor functions are intriguing. Studies on stroke survivors suggest that tVNS paired with movements may promote motor recovery in chronic stroke by inducing neuroplasticity [38]. In particular, tVNS combined to robot-assisted training has been proven to be a safer and potentially effective option for upper limb functional rehabilitation after ischemic stroke [35]. The feasibility and effectiveness of such rehabilitation protocol was assessed in a multiple-sessions, double-blind, semi-randomized, sham-controlled trial. Results supported the benefit of tVNS, reporting a greater clinical improvement in patients that received the real stimulation compared to the sham group [39].

Evidence-based literature suggests that tVNS can modulate not only motor but also cognitive functions [34]. In particular, iVNS has been found to improve word recognition memory in patients with epilepsy; delayed recognition memory performance for emotional and neutral words, when applied during a lexical decision task [20]; socio-cognitive memory processing [40] and emotion recognition performance [41]. tVNS has been associated with changes in cognitive function, especially in executive functions [42] due to an increased activity of brainstem nuclei, and nucleus of the tractus solitarius. Indeed, the nucleus of the tractus solitarius projects to the locus coeruleus and dorsal raphe nucleus, leading to increased circulation of monoaminergic neurotransmitters. Norepinephrine circulation may also increase functional connectivity of the hippocampus, amygdala, and prefrontal cortex, thus facilitating robust episodic memories, increasing attention, alertness, and influencing emotions.

Starting from these evidences the aim of our study was to evaluate the online effects of tVNS on motor and cognitive functions. BB and Go/No-go tests were performed in three different conditions: without tVNS, during tVNS, and in sham condition.

According to the bihemispheric model of motor control [43], each hemisphere controls several mechanisms of voluntary movements. Therefore, loss of motor control mediated by the ipsilateral hemisphere could be, therefore, the main cause of ipsilesional upper limb motor impairments. Hence, unilateral brain damage should affect hemisphere-specific deficits in the ipsilesional upper limb in stroke patients. Moreover, recent findings have demonstrated a strong association between the damaged hemisphere and motor control impairments providing that each hemisphere takes part in a different control mechanism for both upper limbs. Studies suggest that ipsilesional upper limb training improves motor coordination and functional performance not only in the trained side but also in the contralesional, increasing participation in ADL [44]. For this reason, we decided to evaluate not only the contralesional side but also the ipsilesional one.

The BB test without stimulation (Basal condition) showed lower values not only for the contralesional side, but also for the ipsilesional one when compared to normative data (71 bocks), suggesting that the ipsilesional side is definitely “not-healthy”. The results of our study showed an online effect induced by tVNS on gross motor upper limb functions evaluated through BB in a group of stroke patients. In fact, a statistical difference between Basal-Real was found for both contra and ipsilesional side. These results are in line with literature supporting that tVNS can improve motor function in patients with stroke [33].

For stroke patients, the Minimal Detectable Change defined for the BB is of 5.5 blocks for the contralesional side [45]. In the current study, the median value of blocks moved from one side to the other for the ipsilesional side was 42.5 without stimulation, 43 in sham condition and it increased to 48 with tVNS; the difference between Basal-Real conditions was 5.5 and therefore can be considered clinically relevant. Regarding the contralesional side, the statistical difference between Basal-Real, despite significant, was not clinically relevant as it consisted of only 4 blocks.

Since the BB test is able to target the impairment of both the contralesional and ipsilesional upper limbs, our results support that it could be a useful test to measure the impairment of brain areas able to control higher cortical functions. In fact, the execution of the BB provides a measure of the pure motor action, which allows to move the single cube, but also the planning of the gesture, the exploration of space, the choice of the cube, the coordination of the gesture, with the involvement of visuomotor areas and movement planning areas, as well as multiple cortical areas and interhemispheric connections. In stroke patients, the interhemispheric activity is reduced immediately after the acute event [46], even if connections between the two hemispheres tend to improve, during the recovery phase of the motor function, language and visuomotor skills [47].

By dividing left and right hemiparetic patients, our results, even if related to a small number of patients, showed different effects of tVNS on the two populations. Data showed a statistical improvement only for the ipsilesional side in right hemiparetic patients when tVNS was applied. The number of blocks was 42.5 at basal, 50.5 with real stimulation and 46 with sham condition and the difference found can be considered clinically relevant (major than 5.5 blocks).

Evidence from literature suggests that after a brain lesion both hemispheres are able to orient the attention laterally, but only the right hemisphere is able to orient the attention towards the ipsilateral side (right part of body).

Several studies [48], [49] show that in most right-handed individuals, paying attention to stimuli involving language elicits brain activity lateralized to the left hemisphere, whereas paying attention to stimuli involving visuo-spatial processing elicits brain activity lateralized to the right hemisphere.

Considering the link between movement and cognition we focused on selective attention, defined as the ability to maintain goal-directed behavior by responding to target stimuli and suppressing responses to non-target stimuli. We investigated the reaction time on a Go/No-go task in basal condition, in sham condition, and with tVNS. No statistical difference, but a general trend in reduced reaction time was found when comparing the median latencies across the three conditions (basal 581,5 msec; real 492,5 msec; sham 540 msec). Furthermore, any statistical difference was found with respect to the number of errors in the three conditions considered.

Our results are in line with previous literature [50] supporting decreased error rates rather than faster responses in selective attention task during tVNS stimulation. Our patients presented a floor effect at baseline for what concerns the accuracy (number of errors and omissions). Therefore, in our study tVNS modulation effect regarded only a reduction in response time.

From a comparison of mean response time to the selective attention task, even if statistical analysis did not show differences between right and left hemiparetic patients, a major reduction in response time was measured in left hemiparetic patients when compared to right hemiparetic patients. This result is in line with the crucial role of the right hemisphere in visuo-spatial attention.

A single session of tVNS seems to be effective in improving upper limb motor functions in post stroke patients, but no effects are produced on cognitive function, even if a positive trend was detected.

A possible explanation of this discrepancy between the two tests performed could be related to the complexity of the underlying functions. Evidence-based literature suggests that GABA-ergic systems play an important role in the neuromodulation of action control processes [51], and in motor learning [52] with high demanding tasks. While these aspects are specific to the BB test, the Go/No-go seems to rely on a less demanding cognitive effort*.* This study has some limitations. First of all the number of patients recruited is very low and patients are both chronic and acute. Stimulation parameters were maintained fixed and no different parameters were tested on patients. Recent literature evidence suggests that different parameters of tVNS could modulate the degree of VNS-dependent neuroplasticity. Pruitt et al. [53] reported an inverse relationship between stimulation intensity and the motor function recovery; in particular, moderate intensity tVNS (0.8 mA) paired with rehabilitation are able to induce a greater functional recovery than lower (0.4 mA) and higher stimulation intensity (1.6 mA), although the mechanism underlying this relationship was not defined.

## IV. Conclusion

The acute effect produced by a single session of tVNS could be related to short term plasticity mechanism. Probably a change in manual dexterity and no effect on cognition observed after a single session of tVNS could be related to the fact that different mechanism are involved and medium-long term treatment are necessary to improve cognitive task.

Single session results are promising; further studies on more patients and with a pre-post-follow up evaluation are necessary to have clear evidence.

## V. Materials and Methods

*Population*

Ten post stroke patients with the following inclusion/exclusion criteria were enrolled. Inclusion criteria were:

– ischaemic or haemorrhagic unilateral stroke
– unilateral motor upper limb impairment
– no contraindication to tVNS
– ability to give informed consent
– no evidence of cognitive impairment or selective deficit in language, attention and executive domain that might interfere with tasks’ comprehension and execution

Exclusion criteria were:

– bilateral upper limb impairments
– global aphasia that limited the comprehension
– Mini Mental Scale Examination <24

Patients signed the informed consent and were evaluated with a set of clinical scales to evaluate motor and cognitive functions like described in Results section.

*Experimental design*

The effect of online tVNS on motor dexterity and action selection was tested in a single session, single-blind, sham controlled study with a within-subject design in which each participant underwent all the experimental conditions.

Patients received real vs sham tVNS during the execution of the Box and Blocks Test and the Go/No-go task; they were asked to perform both tasks with the non-affected upper limb, regardless of their handedness.

After the global evaluation 2 extra sessions of BB test and Go/No-go were performed in 2 different conditions: a) during a real tVNS and b) during a sham tVNS. The electrode of the tVNS was applied in the patient's cymba and was maintained for 15 minutes prior to start the task. After 15 minutes of real/sham stimulation with the condition maintained, the patient was asked to perform the BB or the Go/No-go task.

The intensity of stimulation was individually set and kept below the pain threshold (mean = 0.8 mA) and it was applied only on the left side to reduce the risk of bradycardia.

In sham tVNS sessions, electrodes were attached in the same location as in real conditions, but the stimulation was delivered at 0 mA. The order of the two conditions (sham/real) was counterbalanced across participants.

The Box and Blocks Test and Go/No-go Test were the primary outcome measures and were performed at baseline and during each stimulation session.

*Statistical Analysis*

Due to the small number of patients enrolled, a nonparametric analysis was conducted. The Friedman test was used to make multiple comparisons between the three conditions (basal, real stimulation, sham). Wilcoxon test, a non-parametric test that compares two paired groups, was employed to detect significant changes between the three conditions considered. Statistical significance was set at 0.05. Statistical analyses were performed with SPSS Statistics (IBM Corporation, Armonk, NY, USA). The population was divided in 2 groups: patients with left and right hemiparesis and the subgroup analysis was performed.

## VI. Supplementary Materials

The description of the device used for the study is presented in this section.
